# Single-cell multi-omics reveal stage of differentiation and trajectory-dependent immunity-related gene expression patterns in human erythroid cells

**DOI:** 10.3389/fimmu.2024.1431303

**Published:** 2024-08-29

**Authors:** Roman Perik-Zavodskii, Olga Perik-Zavodskaia, Saleh Alrhmoun, Marina Volynets, Julia Shevchenko, Kirill Nazarov, Vera Denisova, Sergey Sennikov

**Affiliations:** ^1^ Laboratory of molecular immunology, Federal State Budgetary Scientific Institution Research Institute of Fundamental and Clinical Immunology, Novosibirsk, Russia; ^2^ Clinic of immunopathology, Federal State Budgetary Scientific Institution Research Institute of Fundamental and Clinical Immunology, Novosibirsk, Russia

**Keywords:** erythroid cells, CD71+ erythroid cells, CECs, acute lymphoblastic leukemia, ALL, CITE-seq, scRNA-seq

## Abstract

The role of Erythroid cells in immune regulation and immunosuppression is one of the emerging topics in modern immunology that still requires further clarification as Erythroid cells from different tissues and different species express different immunoregulatory molecules. In this study, we performed a thorough investigation of human bone marrow Erythroid cells from adult healthy donors and adult acute lymphoblastic leukemia patients using the state-of-the-art single-cell targeted proteomics and transcriptomics via BD Rhapsody and cancer-related gene copy number variation analysis via NanoString Sprint Profiler. We found that human bone marrow Erythroid cells express the *ARG1, LGALS1, LGALS3, LGALS9*, and *C10orf54* (VISTA) immunosuppressive genes, *CXCL5, CXCL8*, and *VEGFA* cytokine genes, as well as the genes involved in antimicrobial immunity and MHC Class II antigen presentation. We also found that *ARG1* gene expression was restricted to the single erythroid cell cluster that we termed ARG1-positive Orthochromatic erythroblasts and that late Erythroid cells lose *S100A9* and gain *MZB1* gene expression in case of acute lymphoblastic leukemia. These findings show that steady-state erythropoiesis bone marrow Erythroid cells express myeloid signature genes even without any transdifferentiating stimulus like cancer.

## 1 Introduction

Erythroid cells go through a unique path, in the process of which transiently existing (for just two weeks) (1) nucleated precursor cells of human bone marrow erythrocytes provide local immunoregulation by expressing cytokines, chemokines (2–5) and immunosuppressive enzyme Arginase-1 (6–8), after which they lose their nucleus, and with it the ability to synthesize immunoregulatory molecules. This circumstance makes erythroid cells a unique immunoregulatory population, that is destined to terminally differentiate and that only exists exclusively in the bone marrow in normal condition.

The erythron is represented by 6 successive stages of differentiation: Burst-forming unit-Erythroid (BFU-E), Colony-forming unit-Erythroid (CFU-E), Proerythroblasts (Pro Eb), Basophilic erythroblasts (Baso Eb), Polychromatophilic erythroblasts (Poly Eb), Orthochromatophilic erythroblasts (Ortho Eb) (9, 10), during which a change in the immunoregulatory potential of erythroid cells was observed (6, 11).

Human erythroid cells can be identified by their expression of the genes *GYPA, GYPB, GYPC* and their respective proteins CD235a, CD235b, and CD235c that can be observed from the Proerythroblast stage and onward (12–14); as well as *ALAS2* gene, expression of which can be detected even at the BFU-E stage and onward (15, 16).

Erythroid cells normally make up to 30% of bone marrow mononuclear cells (17), which makes them one of the key populations that take part in the regulation of the bone marrow microenvironment. However, there are conditions that can disrupt erythropoiesis, such as lympho- (18) and myeloproliferative (19–21) diseases that, especially in the blaster crisis phase, lead to the physical displacement of erythron cells by tumor clone cells (22, 23). The occurrence of lympho- and myeloproliferative diseases is caused by chromosomal rearrangements, such as translocations and changes in the set of chromosomes. One such example is the Philadelphia chromosome (Ph) [a product of mutual translocation between chromosomes 9 and 22, t (9, 22)] which is present in 90–95% of cases of chronic myeloid leukemia (24). A change in the set of chromosomes (ploidy) is also often accompanies acute lymphoblastic leukemia (25, 26), and changes in the ploidy of chromosome 21 are especially common (27), as well as genomic changes, such as mutations and changes in the number of copies of proto-oncogenes, cell cycle genes, surveillance of DNA damage and apoptosis (28–32), however, it is usually clonal and does not extend to other bone marrow cells.

**Figure 1:**
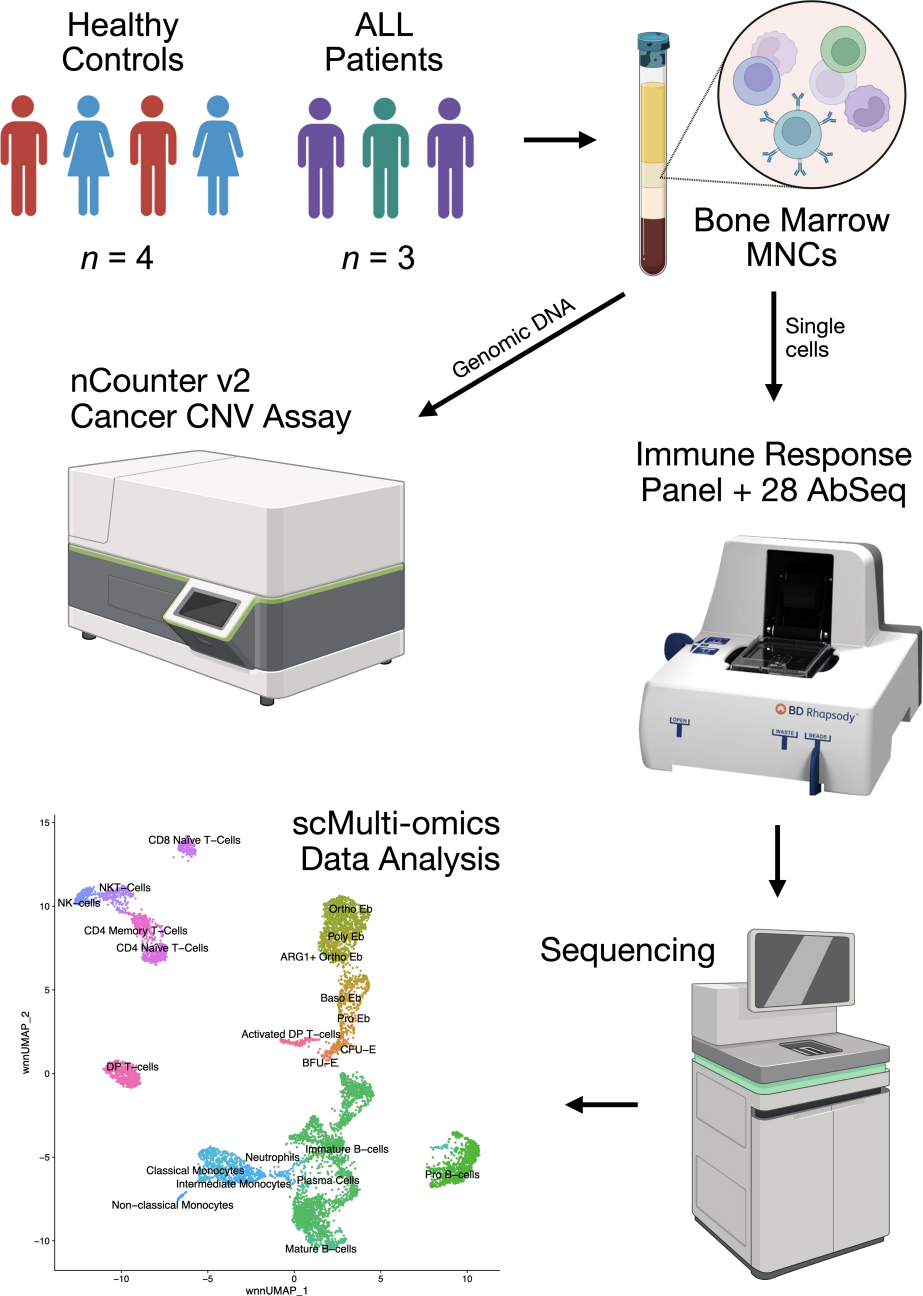
Overview of the experiment, this figure was created via BioRender.

It can be assumed that the very fact of the presence of a tumor clone in the bone marrow will lead to modulation of the immunoregulation provided by erythroid cells in the form of the pressure exerted on them by the tumor cell mass and tumor-expressed cytokines and other immunoregulatory molecules. To test the above-mentioned hypothesis, in this study we performed a multi-omic analysis of bone marrow mononuclear cells from healthy adult donors and mononuclear cells from acute lymphoblastic leukemia using simultaneous single-cell immune transcriptome (397 genes) and surface protein (28 proteins) expression profiling on the BD Rhapsody platform using the Cellular Indexing of Transcriptomes and Epitopes using Next Generation Sequencing (CITE-seq) method, as well as bulk profiling of the number of copies of genes associated with carcinogenesis of the investigated acute lymphoblastic leukemias, using the NanoString Copy Number technology on the Sprint platform (**Figure 1**).

## 2 Materials and methods

### 2.1 Human bone marrow sample collection and processing

We obtained bone marrow samples from both male and female healthy controls (*n* = 4) and male acute lymphoblastic leukemia patients (ICD code C91) (*n* = 3). The healthy study subjects were between the ages of 27 and 48 without any underlying conditions and no clinical evidence of anemia. Acute lymphoblastic leukemia samples were collected during the diagnostic bone marrow biopsy and pre-clinical treatment of the patients.

Acute lymphoblastic leukemia (ALL) patient status at the moment of diagnostic bone marrow biopsy was: ALL patient 1 - 42 years old, first relapse of leukemia, was initially diagnosed with CD19+ ALL and received successful Blinatumomab (clinical-grade anti-CD19 Ab) treatment a year prior; ALL patient 2 - 47 years old, newly-discovered ALL; ALL patient 3 - 27 years old, first relapse of leukemia, was initially diagnosed with CD19+ ALL and received successful Blinatumomab treatment 2 years prior. Patient status one year after the diagnostic bone marrow biopsy: ALL patient 1 - alive, in remission after another round of Blinatumomab treatment; ALL patient 2 - alive; ALL patient 3 - dead, died 4 months after the biopsy procedure due to heart failure.

We collected the bone marrow aspirates (up to 5 mL in volume) into tubes containing EDTA. We isolated bone marrow mononuclear cells using density gradient centrifugation (Ficoll-Paque™, Thermo Fisher Scientific, Waltham, USA) with a density of 1.077 g/mL) at 266 RCF for 30 min in order to remove RBCs.

### 2.2 Single-cell multi-omic analysis

#### 2.2.1 Sample tag and AbSeq cell staining and counting

We incubated mononuclear cells with Sample Tag antibodies to barcode individual samples and 28 AbSeq (CD3:SK7 | CD3E | AHS0033 | Cat#940000, CD4:SK3 | CD4 | AHS0032 | Cat#940001, CD8:SK1 | CD8A | AHS0228 | Cat#940305, CD14:MPHIP9 | CD14 | AHS0037 | Cat#940005, CD15 | FUT4 | AHS0196 | Cat#940274, CD16:B73.1 | FCGR3A_FCGR3B | AHS0242 | Cat#940314, CD19:HIB19 | CD19 | AHS0161 | Cat#940247, CD1a | CD1A | AHS0067 | Cat#940063, CD30 | TNFRSF8 | AHS0114 | Cat#940103, CD33:WM53 | CD33 | AHS0044 | Cat#940031, CD34:563 | CD34 | AHS0191 | Cat#940367, CD36 | CD36 | AHS0135 | Cat#940224, CD38:HIT2 | CD38 | AHS0022 | Cat#940013, CD40 | CD40 | AHS0117 | Cat#940049, CD44:L178 | CD44 | AHS0167 | Cat#940251, CD45RA: HI100 | PTPRC | AHS0009 | Cat#940011, CD45RO | PTPRC | AHS0036 | Cat#940022, CD49d | ITGA4 | AHS0063 | Cat#940059, CD56:NCAM16.2 | NCAM1 | AHS0019 | Cat#940007, CD64:MD22 | FCGR1A | AHS0180 | Cat#940262, CD71 | TFRC | AHS0197 | Cat#940275, CD79b:CB3-1 | CD79B | AHS0153 | Cat#940239, CD86:BU63.224 | CD86 | AHS0245 | Cat#940315, CD95 | FAS | AHS0023 | Cat#940037, CD117:YB5.B8 | KIT | AHS0064 | Cat#940051, CD235a_b | GYPA_GYPB | AHS0048 | Cat#940040, CCR7 | CCR7 | AHS0273 | Cat#940394, HLA-DR | CD74 | AHS0035 | Cat#940010, BD Biosciences) antibodies for surface protein expression profiling for 30 minutes at room temperature according to the manufacturer’s recommendations (“Single Cell Labelling with BD AbSeq Ab-Oligos (1 to 40 plex)”).

After three washing cycles, cells were stained with Calcein according to the BD Rhapsody Single-Cell Analysis System User Guide. Calcein-positive cells were counted using the Attune NxT flow cytometer as events/uL. Cells were then pooled together in equal proportions and resuspended in a cold sample buffer to a final concentration of 15 cells/µl for loading onto a BD Rhapsody Cartridge. The number of cells loading into the cartridge was visually validated using the In Cell Analyzer 6000 as mean Calcein-positive cells in 5 fields of view (FOV)/175 (microwells per FOV) * 200000 (total number of microwells per cartridge).

#### 2.2.2 CITE-seq library preparation and sequencing

We performed single-cell capture and cDNA library preparation using the BD Rhapsody Express Single-Cell Analysis System (BD Biosciences), according to the manufacturer’s instructions (mRNA Targeted, Sample Tag, and BD AbSeq Library Preparation Protocol). Briefly, we captured single cells in the BD Rhapsody cartridge, added magnetic beads for poly-A based mRNA capture, lysed the cells, performed reverse transcription of the poly-A captured mRNA, AbSeq and Sample Tag on the magnetic beads, denaturated the Sample Tag and AbSeq from the beads, performed Sample Tag and AbSeq PCR 1, treated the beads with Exonuclease I, amplified the on-bead cDNA using the Human Immune Response Primer Panel (mRNA for short) (#633750, BD Biosciences), containing 399 primer pairs, targeting 397 different genes, collected the mRNA panel PCR1 products.

We purified the resulting mRNA, AbSeq, and Sample Tag PCR1 products using AMPure XP magnetic beads (A63880, Beckman Coulter, Brea, California, United States) and separated the respective mRNA panel, AbSeq, and Sample Tag products based on the amplicon size. We further amplified the purified mRNA and Sample Tag PCR1 products in a semi-nested PCR2 for an increase in specificity of the transcript detection and purified the resulting PCR2 products using AMPure XP magnetic beads. We assessed the PCR2 product concentrations by Qubit 4 (High-Sensitivity dsDNA Kit, Thermo Fisher) and normalized the final products to 4.5 ng/μL for the mRNA panel library and 1.0 ng/μL for the AbSeq and Sample Tag library and performed a final round of amplification using indexes for Illumina sequencer to prepare the final libraries. We quantified the final libraries using Qubit 4 and pooled them (~40/57/3% mRNA/AbSeq/Sample Tag ratio, estimated read/cell: 20000 (mRNA, deep sequencing read count quantity), 28000 (AbSeq, 1000 reads per AbSeq) and 1200 (Sample Tag)) to the final concentration of 5 nM. The final pooled libraries were sequenced (R1 = 71, R2 = 51, 1300 million clusters, S1 flow cell) on a NovaSeq 6000 sequencer (Illumina, San Diego, California, United States).

#### 2.3.3 Raw data processing

We processed the FASTQ files obtained from sequencing using the BD Rhapsody pipeline v1.10 (BD Biosciences). The pipeline removed read pairs with low quality based on their read length, mean base quality score, and highest single-nucleotide frequency, analyzed remaining high-quality R1 reads to identify cell label and unique molecular identifier (UMI) sequences, aligned the remaining high-quality R2 reads to the reference mRNA and AbSeq panel sequences using Bowtie2, collapsed reads with the same cell label, the same UMI sequence and the same gene into a single molecule, adjusted the obtained counts by error correction algorithms, namely, recursive substitution error correction (RSEC) and distribution-based error correction (DBEC) to correct for sequencing and PCR errors, estimated cell counts using the second derivative analysis to filter out noise cell labels, observed one inflection point, and considered cell labels after that point to be noise labels. Then, the pipeline used molecular barcoded oligo-conjugated Sample Tag antibodies (single-cell multiplexing kit, BD Biosciences) to demultiplex the samples and filter out the cell multiplets. The pipeline called 10536 single cells (~1500 cells per sample, *n* = 7) and output combined gene and surface protein expression matrices for each sample. Sequencing metrics showed sequencing saturation of 98% and adjusted DBEC sequencing depth of 8.1, which is considered deep sequencing for BD Rhapsody libraries.

#### 2.2.4 Multi-omic data analysis via Seurat WNN

We manually split the combined gene and surface protein expression matrices for each sample into gene and surface protein expression matrices respectively and analyzed them using Seurat WNN (Weighted Nearest Neighbors) (33). We imported gene expression matrices, created Seurat objects for each sample, added AbSeq surface protein expression data to each object as ADT (antibody-derived tag) data, merged the individual objects, and subjected them to a quality control procedure (nCount_RNA < 3200, nCount_ADT < 65000). Then, we found the most variable genes in expression for the merged object (we used all 397 genes). The merged gene expression matrix was normalized using the SCTransform v2 (SCT) package (34) of the R programming language taking into account the selected variable genes. For the SCT-normalized gene expression matrix, we performed PCA (principal component analysis) dimensionality reduction and corrected the batch effect using the Harmony package (35) of the R programming language. The merged ADT matrix was normalized using the Centered Log-ratio (CLR) normalization method taking into account all 28 surface proteins. For the CLR-normalized ADT matrix, we performed PCA dimensionality reduction and corrected the batch effect using the Harmony package of the R programming language. We then performed Weighted Nearest Neighbors Uniform Manifold Approximation and Projection (WNN UMAP) multi-omic dimensionality reduction using 22 Harmony-corrected gene expression principal components and 18 Harmony-corrected ADT principal components, found multi-omic neighbors and clusters (resolution = 1.5).

We manually annotated the resulting clusters using their surface protein and gene expression data: Activated DP (double positive) T−cells: CD4+ CD8+ CD71+; DP T−cells: CD4+ CD8+; CD4 Memory T−Cells: CD4+ *CCR7*−; CD4 Naïve T−Cells: CD4+ *CCR7*+; CD8 Naïve T−Cells: CD8+ *CCR7*+; NKT−Cells: CD8+ CD56+ *NKG7*+; NK−cells: CD16+ CD56+ *NKG7*+; Non−classical Monocytes: CD16+; Intermediate Monocytes: CD14dim CD64dim; Classical Monocytes: CD14+ CD64+; Neutrophils: *AZU1*+; Plasma Cells: CD19+ *JCHAIN*+; Mature B−cells: CD19+ *IGHM*+; Immature B−cells: CD19+ CD38+; Pro B−cells: CD19+ CD34+; Nucleated Erythroid cells: *ALAS2+ SNCA+ SLC25A37+*. We then created the *DimPlot* of the clusters colored by the bio-group (ALL 1, ALL2, ALL 3, Normal BM (bone marrow)) via Seurat.

Leukemia clusters for each ALL sample were found as mostly (>90%) represented by a single sample and were identified as follows: ALL 1 – Pro B-cells; ALL 2 – Double Positive (DP) T-cells, and Activated DP T−cells; ALL 3 – Pre-B-cells. We then exported CLR-normalized surface protein expression values for the ALL clusters as.csv via the *AverageExpression* function and created the heat map of the averaged log2-transformed expression values via bioinfokit (36).

#### 2.2.4 Erythroid cell sub-clustering

To study human bone marrow Erythroid cells with greater precision, we subclustered the “Nucleated Erythroid cells” cluster from the total mononuclear cells using the *subset* function. Then, we found the most variable genes in expression for the Erythroid cells (we used all 397 genes). The Erythroid cell gene expression matrix was normalized using the SCTransform v2 (SCT) package of the R programming language taking into account the selected variable genes. For the SCT-normalized gene expression matrix, we performed PCA dimensionality reduction and corrected the batch effect using the Harmony package of the R programming language. The Erythroid cell ADT matrix was normalized using the Centered Log-ratio (CLR) normalization method taking into account all 28 surface proteins. For the CLR-normalized ADT matrix, we performed PCA dimensionality reduction and corrected the batch effect using the Harmony package of the R programming language. We then performed Weighted Nearest Neighbors Uniform Manifold Approximation and Projection (WNN UMAP) multi-omic dimensionality reduction using 26 Harmony-corrected gene expression principal components and 9 Harmony-corrected ADT principal components, found multi-omic nucleated erythroid cell neighbors and clusters (resolution = 0.6), and created a *DimPlot* of the clusters.

We manually identified Erythroid cell clusters using their surface protein and gene expression as follows: Burst-forming unit–erythroid (BFU–E): CD71+ CD235− CD34+ CD38+ CD36− CD49d+ CD44+ *ITGA4+ CD36*− *CD44*+ *CD34+ CD38+*; Colony-forming unit–erythroid (CFU−E): CD71+ CD235− CD34− CD38+ CD36+ CD49d+ CD44+ *ITGA4+ CD36+ CD44+ CD34− CD38+*; Proerythroblast (Pro Eb): CD71+ CD235+ CD36+ CD49d+ CD44+ *ITGA4+ CD36+ CD44+*; Basophilic erythroblast (Baso Eb): CD71+ CD235+ CD36+ CD49d+ CD44− *ITGA4+ CD36+ CD44−*; Polychromatophilic erythroblast (Poly Eb): CD71+ CD235+ CD36+ CD49d− *ITGA4− CD36−*; Orthochromatophilic erythroblast (Ortho Eb): CD71+ CD235+ CD36− CD49d− *ITGA4− CD36−*; and a newly-found *ARG1+* Orthochromatophilic erythroblast (ARG1+ Ortho Eb): *ARG1*+ CD71+ CD235+ CD36− *ITGA4− CD36−*. We then created the *DotPlot* of the Erythroid cell marker genes and surface proteins via Seurat and created the Stacked bar plot of the Erythroid cell cluster percentages via ggplot2.

We replaced the original metadata of the “Nucleated Erythroid Cell” cluster with the Erythroid cell sub-clustering metadata using the *paste(Idents())* function and created the *DimPlot* of all bone marrow cell clusters via Seurat and the Stacked bar plot of the cell percentage per cluster via ggplot2.

#### 2.2.5 Erythroid cell trajectory analysis via Slingshot and TradeSeq

Next, we employed the Slingshot (37) and TradeSeq (38) libraries for the R programming language to infer the Erythroid cell differentiation trajectory. We used multi-omic WNN UMAP embeddings and WNN-multi-omic clusters to infer cell lineages via the *getLineages* function, performed the *getCurves* function on the lineages, and performed the *fitGAM* function on the lineages using SCT-normalized gene expression values. We observed two lineages that were split at the Poly Eb stage. We then used the Wald test to test for the genes that drove the branch division and observed that the *ARG1* gene was solely responsible for the division. We then created gene expression and curve plots to depict the branch division driving gene *ARG1* via ggplot2.

#### 2.2.6 Erythroid cell gene expression analysis

Then, we performed Erythroid cell gene expression hierarchical clustering for the WNN-multi-omic clusters. We exported the SCT-normalized gene expression values for the Erythroid cell clusters as.csv via the *AverageExpression* function and then performed the Z-score data standardization and hierarchical clustering via bioinfokit (36). We observed the stage of differentiation-defined gene expression clusters, recreated their hierarchical clustering-defined order of the immunoregulatory genes in Seurat, and created a *DotPlot* of the genes via Seurat.

We tested the stage of differentiation-defined gene expression cluster genes along with the universally expressed erythroid cell *ALAS2, SNCA, GAPDH, SLC25A37, HLA-A, TFRC* (CD71), and *GYPA* (CD235a) genes for overrepresentation in the Gene Ontology Biological Process terms via GSEApy (39), and we considered *q*-values < 0.01 significant. We created a DimPlot of the Erythroid cell immunoregulatory genes in the context of all bone marrow cell populations via *DotPlot*.

Then, we performed inter-cluster differential gene expression using the Wilcoxon test with biological and statistical significance criteria of log2(Fold Change) > 1.0 or log2(Fold Change) < −1.0 and q-value < 0.005 via the *FindMarkers* function. We only considered genes to be differentially expressed in ALL if they were up- or down-regulated in every single pairwise comparison: ALL 1 vs Normal BM, ALL 2 vs Normal BM, ALL 3 vs Normal BM. We created the *DotPlot* of the differentially expressed genes in Seurat and created the Stacked bar plot of the log2(Fold Change) values per pairwise comparison in GraphPad Prism 10.2.3.

### 2.3 Copy number variation analysis

#### 2.3.1 Magnetic separation

We performed magnetic separation of the bone marrow mononuclear cells using a magnetic stand, a magnet (Miltenyi Biotec, 130-042-102, Bergisch Gladbach, Cologne, Germany), and either CD8 MicroBeads (130-045-201, Miltenyi Biotec, Bergisch Gladbach, Cologne, Germany) for ALL 2 or CD19 MicroBeads (130-050-301, Miltenyi Biotec, Bergisch Gladbach, Cologne, Germany) for ALL 1 and ALL 3 according to the manufacturer’s protocols.

#### 2.3.2 Genomic DNA extraction

We isolated total DNA from the enriched ALL cells after their magnetic separation and normal bone marrow mononuclear cells using a Genomic DNA Purification Kit (24700, Norgen Biotek, Thorold, Canada), measured the concentration of the DNA on a Qubit 4 using the High Sensitivity dsDNA kit (Q32851, Thermo Fisher Scientific, Waltham, USA).

#### 2.3.3 Alu1 restriction digest of the genomic DNA

We performed a 2h restriction digest of the 500 ng of the isolated DNA for each sample using the Alu1 restriction enzyme supplied with the nCounter v2 Cancer CN Assay at 37°C, measured the concentration of the digested DNA on a Qubit 4, and diluted the digested DNA to a concentration of 40 ng/μL using nuclease-free water. We froze the diluted total DNA at −80°C until the cancer-related gene CNV profiling.

#### 2.3.4 NanoString v2 cancer CNV assay

We performed cancer-related gene CNV profiling with the help of the Nanostring nCounter SPRINT Profiler analytical system using 200 ng of the restricted DNA. We used an nCounter v2 Cancer CN Assay panel (CNV-CAN2-24, NanoString) to analyze the restricted DNA. The nCounter v2 Cancer CN Assay consists of 87 target cancer-related genes, 54 invariant DNA segments spanning multiple chromosomes for data normalization, and 8 negative and 6 positive controls, each DNA segment was profiled using 3 distinct probes. The samples were subjected to a 20h hybridization reaction at 65°C, where 5 μL of the restricted DNA was combined with 3 μL of nCounter Reporter probes, 7 μL of DEPC-treated water, 10 μL of hybridization buffer, and with 5 μL of nCounter capture probes (total reaction volume = 30 μL). After the hybridization of the probes, we added 10 uL of the hybridization buffer and counted the number of target molecules on the Nanostring nCounter SPRINT Profiler analytical system. We then extracted the data from the SPRINT Profiler, performed data QC and normalization in nSolver 4, and exported the normalized data as a.tsv file.

We then performed CNV analysis according to the manufacturer’s guidelines (CNV Hybridization Protocol, MAN-10093-01, NanoString). In brief, we assessed the assay linearity for all samples using the Coefficient of Determination (r^2^ was > 0.994 for all samples), calculated median counts for the invariant DNA segments, calculated the normalization factor, normalized all samples using the normalization factor, averaged the probe count for each gene, and divided each gene average probe count by the normal bone marrow (control sample) counts. As chromosome counts were required for the next step of the analysis, karyotype analysis of the ALL samples was conducted by the “Regional Center of High Medical Technologies” (Novosibirsk, Russia). We then multiplied the division product of the average probe counts by the number of chromosomes present in the genome and rounded up the product to obtain the gene copy numbers.

## 3 Results

### 3.1 Multi-omic characteristic of ALLs

**Figure 2:**
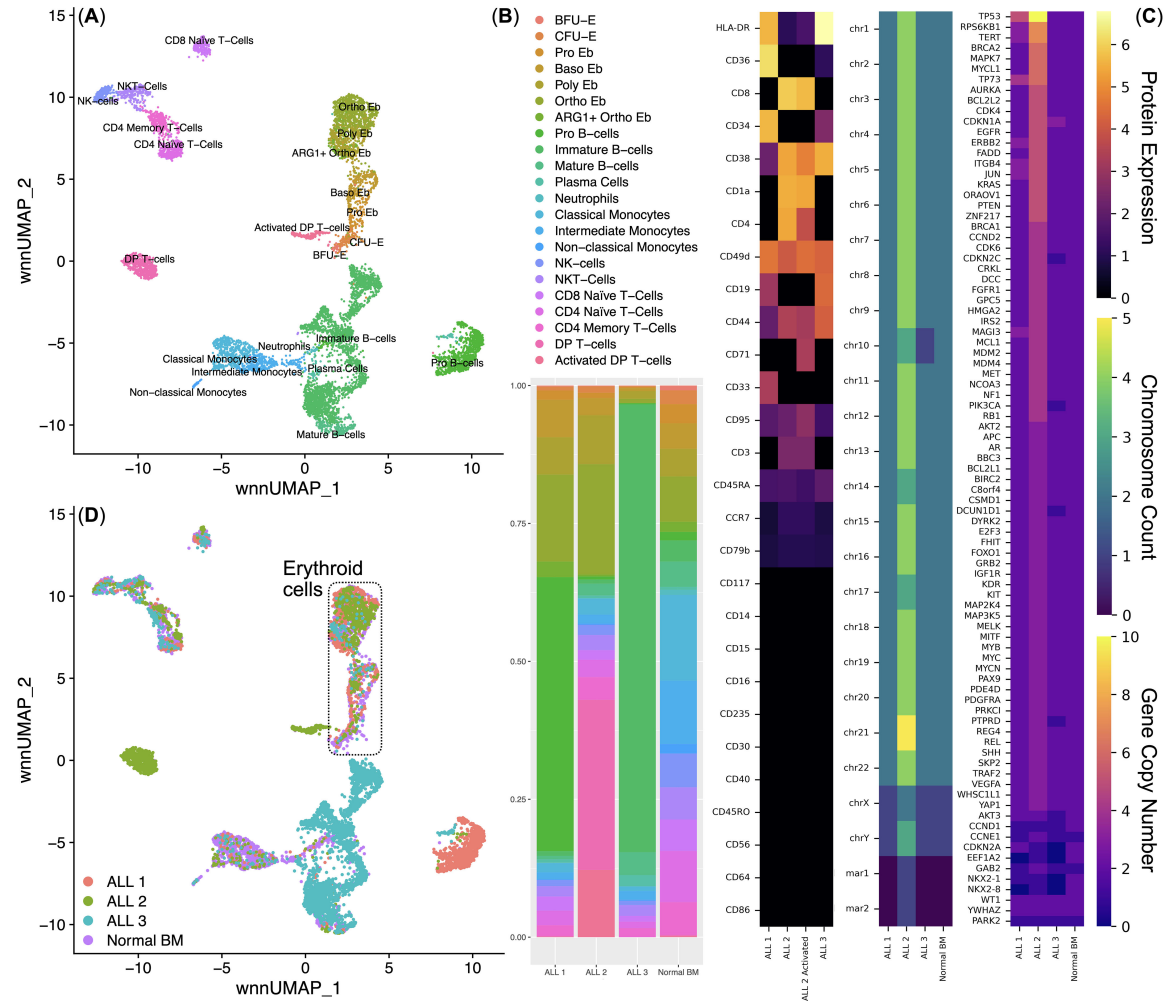
Integrated analysis of the normal (*n* = 4) and acute lymphoblastic leukemia (*n* = 3) bone marrow mononuclear cell single-cell immune transcriptome and surface protein data. **(A)** UMAP plot of the clusters; **(B)** Stacked bar plot of the percentages of cells per cluster per bio-group, clusters are color-labeled in accord with the subFig A; **(C)** multi-omic characterization of ALL samples - CLR-normalized surface protein expression, chromosome and gene copy numbers; **(D)** UMAP plot of the bone marrow mononuclear cell clusters, clusters are color-labeled in accord with the bio-groups (ALL 1, ALL 2, ALL 3, Normal BM.

We performed a multi-omic analysis of bone marrow mononuclear cells from healthy donors (*n* = 4) and patients with ALL (*n* = 3) using the BD Rhapsody single-cell multi-omic analysis method (379 genes of the immune transcriptome and 28 surface proteins) and the Copy Number Variation NanoString analysis method Sprint (nCounter v2 Cancer CN panel) to comprehensively understand the phenotype of the studied ALLs and their influence on erythroid cells of the human bone marrow. We conducted an unbiased clustering analysis of bone marrow mononuclear cells and found 22 clusters (**Figure 2A**), corresponding to normal and ALL cell populations. Clusters for each ALL sample were detected when analyzing the proportions of cells in the bone marrow: ALL 1 – Pro B-cells, ALL 2 – Double Positive (DP) T-cells and Activated DP T−cells, ALL 3 – Pre-B-cells, which was in accordance with the initial clinical assessment (**Figure 2B**).

Before the direct analysis of the influence of ALL on the erythroid cells of the bone marrow, we conducted a multi-omic characterization of the studied ALLs (analysis of the surface proteome, karyotyping, copy number variation) (**Figure 2C**).

ALL 1 expressed surface proteins CD19, CD34, CD33, CD36, CD44, CD45RA, CD49d, CD95, and HLA-DR; had a normal set of chromosomes, had a changed karyotype 46,XY,der(4)?t(4;15),del(11)(p11),der(15)der(19)der(20); had an increased number of copies of the *MAGI3, JUN, ITGB4, ERBB2, TP73, TERT, RPS6KB1*, and *TP53* genes, and also had a decreased number of copies of the *CCND1, EEF1A2, NKX2-1*, and *NKX2-8* genes.

ALL 2 expressed surface proteins CD4, CD8, CD1a, CD38, CD44, CD45RA, CD49d, CD79b, CD95, CCR7, HLA-DR, and additionally expressed CD71 in a cluster of activated tumor T-cells; had an uneven tetraploid set of chromosomes, had a changed karyotype XXYY,+Y,-10,-14,-17,+20,+21,+mar1,+mar2; had an increased number of copies of almost all studied genes, except for *CCND1, EEF1A2, NKX2-1*, and *NKX2-8*.

ALL 3 expressed surface proteins CD19, CD38, CD44, CD45RA, CD49d, CD95, and HLA-DR; had an incomplete diploid set of chromosomes, had an altered karyotype 45,XY,der(3),?i(9)(q10),der(9),-10,der(14),der(15); had an increased number of copies of the CDKN1A gene, and also had a decreased number of copies of the *CDKN2C, PIK3CA, DCUN1D1, PTPRD, AKT3, CCND1, EEF1A2, NKX2-1*, and *NKX2-8* genes. Reduction in the number of copies of *CCND1, EEF1A2, NKX2-1*, and *NKX2-8* genes was conservative for all examined ALLs.

The heterogeneity of the studied ALLs made it possible to analyze ALL as a pathological process affecting Erythroid cells: the diagnosis of C91 “Acute Lymphoblastic Leukemia” was an invariable common factor of the studied samples, and the multiple differences in the nature of the studied ALLs covered most of the possible variations in ALL: T- and B-cell ALL with different phenotypes, the presence or absence of chromosome ploidy changes, as well as the presence of CNVs of different genes. Then, from all clusters (**Figure 2D**), we sub-clustered Erythroid cells for their detailed analysis.

### 3.2 Multi-omic analysis of human bone marrow erythroid cells

**Figure 3:**
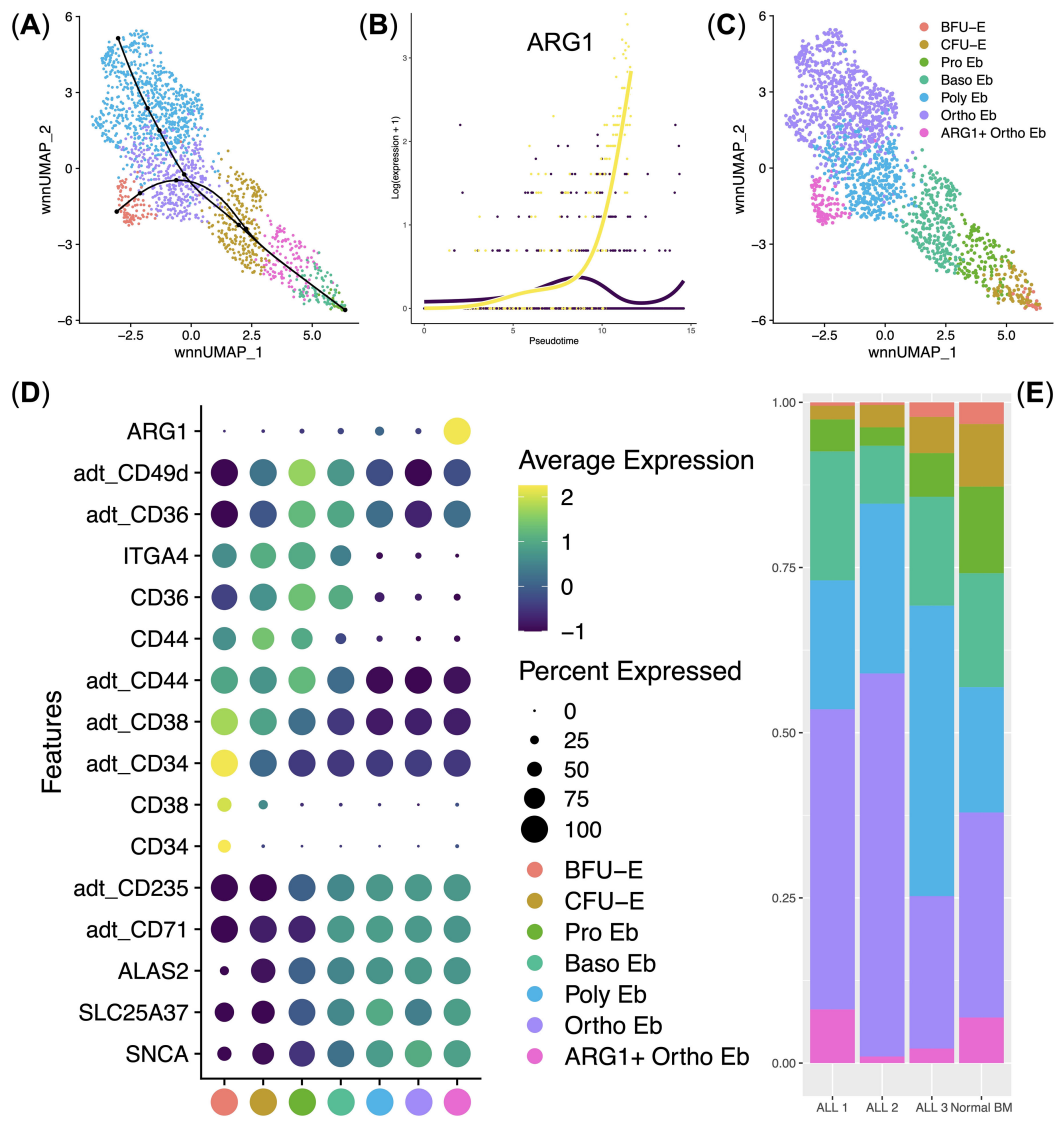
Analysis of normal (*n* = 4) and ALL (*n* = 3) Bone Marrow Erythroid cell immune transcriptome and surface protein expression data. **(A)** UMAP plot of the clusters with the differentiation trajectories overlaid on top; **(B)** Scatter plot of the pseudotime-driving gene *ARG1*; **(C)** UMAP plot of the Erythroid cell clusters/stages of differentiation; **(D)** Dot plot of the cluster-specific gene and protein expression signatures, adt_ - AbSeq antibody-derived tag, mean marker expression values were Z-score transformed, the deep purple color represents the lowest marker expression whereas the yellow color represents the maximum marker expression, dot size represents the percentage of Erythroid cells positive for the marker, clusters are color-labeled in accord with the subFig C; **(E)** Stacked bar plot of the percentages of Erythroid cells per cluster per bio-group, clusters are color-labeled in accord with the subFig C.

Next, we performed an analysis of the subclustered Erythroid cells. We first performed WNN UMAP dimensionality reduction and clustering of Erythroid cells, inferred and found two Erythroid cell differentiation trajectories splitting at the polychromatophilic erythroblasts stage using Slingshot (**Figure 3A**), found trajectory driving-genes (**Figure 3B**) and identified the clusters using their gene and surface protein expression (**Figures 3C, D**). During our clustering analysis, we found all stages of erythroid cell differentiation: burst-forming units (BFU-E), colony-forming units (CFU-E), proerythroblasts (Pro Eb), basophilic erythroblasts (Baso Eb), polychromatophilic erythroblasts (Poly Eb), orthochromatophilic erythroblasts (Ortho Eb), as well as the newly-found *ARG1* gene expressing orthochromatophilic erythroblasts (ARG1+ Ortho Eb) (**Figures 3C, D**).

Immunosuppressive enzyme Arginase 1-encoding gene *ARG1* was almost uniquely expressed in ARG1+ Ortho Eb cluster among both normal and ALL human bone marrow Erythroid cells (**Figure 3D**). Moreover, *ARG1* was the sole trajectory-driving gene that split the Poly Eb into either *ARG1*-negative Ortho Eb or *ARG1*-positive ARG1+ Ortho Eb (**Figure 3B**). We also observed that ALL erythroid cell cluster proportions were similar to those of the normal bone marrow erythroid cell cluster percentages – i.e., no block of Erythroid cells differentiation was observed in any of the studied ALL samples (**Figure 3**). We also performed differential regulon activity analysis by pySCENIC between the Ortho Eb and ARG1+ Ortho Eb but found no significantly differentially-activated transcription factors (See **Supplementary Figure 1**).

**Figure 4:**
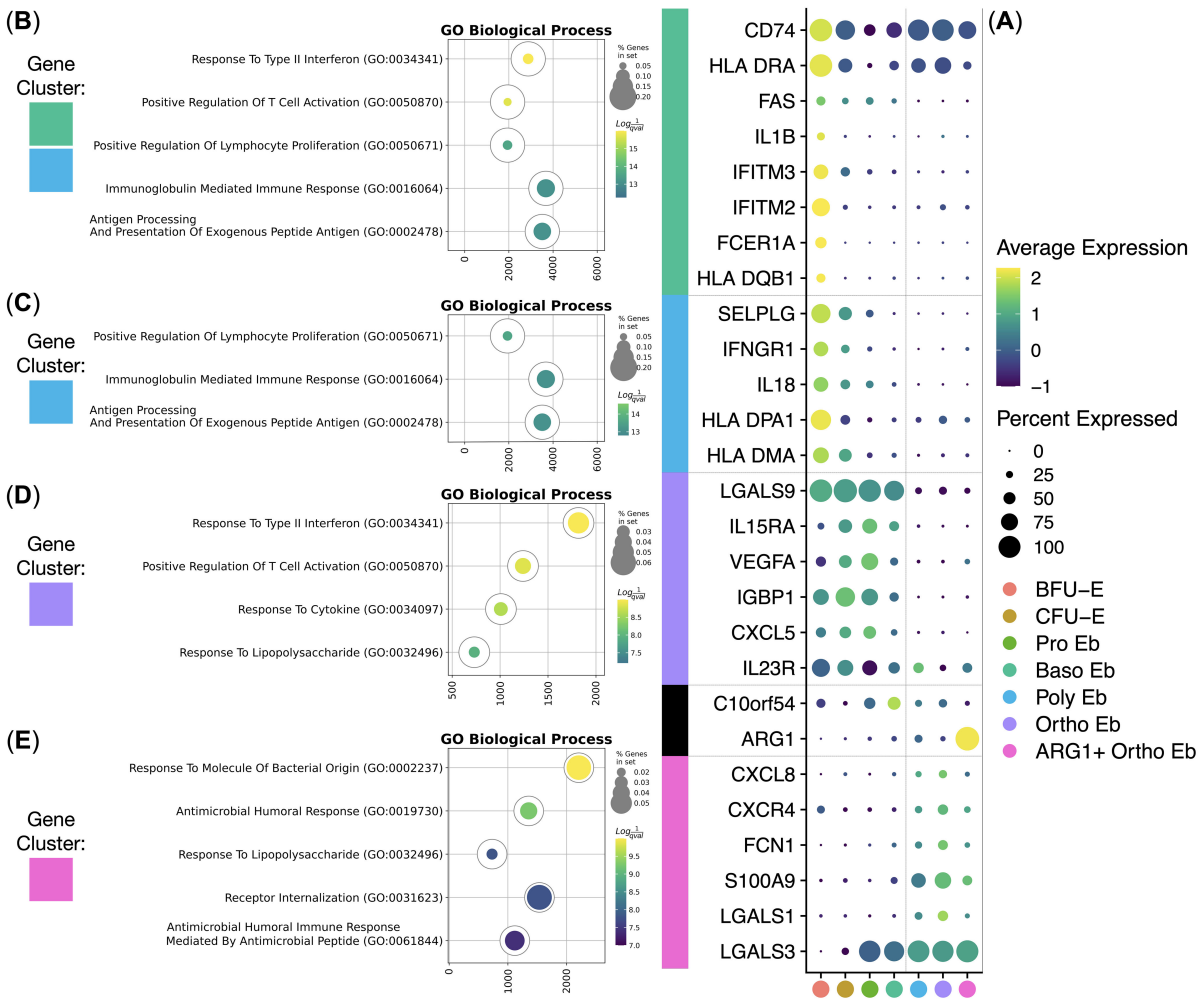
Analysis of healthy human adult bone marrow Erythroid Cells’ (*n* = 4) immunity-related gene clusters. **(A)** Dot plot of the cluster-forming genes, mean marker expression values were Z-score transformed, the deep purple color represents the lowest marker expression whereas the yellow color represents the maximum marker expression, dot size represents the percentage of Erythroid cells positive for the marker, clusters are color-labeled in accord with the **Figure 3C**, clusters are split into early and late by a vertical dotted line, genes are split into clusters by horizontal dotted lines; **(B–E)** Gene Ontology Biological Process overrepresentation analysis of the genes with the detected expression in healthy human adult bone marrow Erythroid Cells. Yellow color corresponds to the lowest *q*-value, purple color corresponds to the highest *q*-value, and the dot size reflects the percentage of genes in the analysis from the full set of genes in the Gene Ontology Biological Process database, each GO BP overrepresentation analysis is color-labeled in accord with the subFig A.

Then, we carried out hierarchical clustering of the averaged gene expression values per normal Erythroid cell cluster and observed stage-of-differentiation-dependent immunity-related gene expression patterns (**Figure 4A**). We observed 4 main gene expression clusters: BFU-E gene cluster, CFU-E gene cluster, early Erythroid cell gene cluster, and late Erythroid cell gene cluster.

The BFU-E gene cluster included *HLA-DMA, HLA-DPA1, IL18, IFNGR1, SELPLG, HLA-DQB1, FCER1A, IFITM2, IFITM3, IL1B*, and *FAS* genes that were overrepresented in the “Antigen Processing And Presentation Of Exogenous Peptide Antigen”, “Peptide Antigen Assembly With MHC Class Il Protein Complex” and “MHC Class Il Protein Complex Assembly” GO BP terms (**Figure 4B**, **Table 1**).

**Table 1:** Gene Ontology Biological Process overrepresentation analysis of the immunity-related genes with the detected expression in human adult bone marrow BFU-E.

Gene Ontology Biological Process Term	Overlap	*Q*-value	Score	Genes
Response To Type II Interferon	6/80	0,000000	2875	*IFITM3, CD74, IFITM2, GAPDH, HLA-DPA1, SNCA*
Positive Regulation Of T Cell Activation	6/107	0,000000	1942	*HLA-DMA, TFRC, IL1B, HLA-DRA, HLA-A, HLA-DPA1*
Positive Regulation Of Lymphocyte Proliferation	5/74	0,000001	1939	*CD74, TFRC, IL1B, IL18, HLA-DPA1*
Immunoglobulin Mediated Immune Response	4/30	0,000002	3678	*CD74, HLA-DMA, HLA-DRA, HLA-DPA1*
Antigen Processing And Presentation Of Exogenous Peptide Antigen	4/31	0,000002	3514	*HLA-DMA, HLA-DRA, HLA-A, HLA-DPA1*
Response To Cytokine	5/125	0,000009		*IFITM3, CD74, IFITM2, IL1B, SNCA*
Positive Regulation Of Type II Interferon Production	4/58	0,000017	1500	*IL1B, IL18, HLA-A, HLA-DPA1*
MHC Class II Protein Complex Assembly	3/14	0,000017	7988	*HLA-DMA, HLA-DRA, HLA-DPA1*
Peptide Antigen Assembly With MHC Class II Protein Complex	3/14	0,000017	7988	*HLA-DMA, HLA-DRA, HLA-DPA1*
Positive Regulation Of Cytokine Production	6/320	0,000020	429	*CD74, IL1B, IL18, HLA-A, GAPDH, HLA-DPA1*
Positive Regulation Of T Cell Proliferation	4/65	0,000020	1287	*TFRC, IL1B, IL18, HLA-DPA1*
Peptide Antigen Assembly With MHC Protein Complex	3/18	0,000028	3581	*HLA-DMA, HLA-DRA, HLA-DPA1*

The CFU-E gene cluster was in fact a truncated BFU-E gene cluster that included *SELPLG, HLA-DQB1, FCER1A, IFITM2, IFITM3, IL1B*, and *FAS* genes that were also overrepresented in the “Antigen Processing And Presentation Of Exogenous Peptide Antigen”, “Peptide Antigen Assembly With MHC Class Il Protein Complex” and “MHC Class Il Protein Complex Assembly” GO BP terms (**Figure 4C**, **Table 2**).

**Table 2:** Gene Ontology Biological Process overrepresentation analysis of the immunity-related genes with the detected expression in human adult bone marrow CFU-E.

Gene Ontology Biological Process Term	Overlap	*Q*-value	Score	Genes
Immunoglobulin Mediated Immune Response	4/30	0,000001	5932	*CD74, HLA-DMA, HLA-DRA, HLA-DPA1*
Antigen Processing And Presentation Of Exogenous Peptide Antigen	4/31	0,000001	5672	*HLA-DMA, HLA-DRA, HLA-A, HLA-DPA1*
Positive Regulation Of T Cell Activation (GO:0050870)	5/107	0,000001	2024	*HLA-DMA, TFRC, HLA-DRA, HLA-A, HLA-DPA1*
MHC Class II Protein Complex Assembly	3/14	0,000008	7988	*HLA-DMA, HLA-DRA, HLA-DPA1*
Peptide Antigen Assembly With MHC Class II Protein Complex	3/14	0,000008	7988	*HLA-DMA, HLA-DRA, HLA-DPA1*
Positive Regulation Of Lymphocyte Proliferation	4/74	0,000012	1775	*CD74, TFRC, IL18, HLA-DPA1*
Peptide Antigen Assembly With MHC Protein Complex	3/18	0,000012	5564	*HLA-DMA, HLA-DRA, HLA-DPA1*
Response To Type II Interferon	4/80	0,000012	1602	*CD74, GAPDH, HLA-DPA1, SNCA*
Immunoglobulin Production Involved In Immunoglobulin-Mediated Immune Response	3/19	0,000012	5158	*HLA-DMA, HLA-DRA, HLA-DPA1*
Antigen Processing And Presentation Of Exogenous Peptide Antigen Via MHC Class II	3/26	0,000030	3354	*HLA-DMA, HLA-DRA, HLA-DPA1*
Antigen Processing And Presentation Of Peptide Antigen Via MHC Class II	3/28	0,000034	3035	*HLA-DMA, HLA-DRA, HLA-DPA1*
Positive Regulation Of Cytokine Production	5/320	0,000064	459	*CD74, IL18, HLA-A, GAPDH, HLA-DPA1*

The early Erythroid cell gene cluster spanned BFU-E, CFU-E, Pro Eb, and Baso Eb, and included *IL23R, CXCL5, IGBP1, VEGFA, IL15RA*, and *LGALS9* genes that were overrepresented in the “Response To Type II Interferon” and “Response To Lipopolysaccharide” GO BP terms (**Figure 4D**, **Table 3**).

**Table 3:** Gene Ontology Biological Process overrepresentation analysis of the immunity-related genes with the detected expression in human adult bone marrow early Erythroid cells.

Gene Ontology Biological Process Term	Overlap	*Q*-value	Score	Genes
Response To Type II Interferon	4/80	0,000090	2875	*IL23R, LGALS9, GAPDH, SNCA*
Positive Regulation Of T Cell Activation	4/107	0,000145	1942	*TFRC, IL23R, HLA-A, LGALS9*
Response To Cytokine	4/125	0,000180	1939	*IGBP1, IL23R, LGALS9, SNCA*
Response To Lipopolysaccharide	4/159	0,000354	3678	*IL23R, LGALS9, CXCL5, SNCA*
Negative Regulation Of Cysteine-Type Endopeptidase Activity Involved In Apoptotic Process	3/49	0,000423	3514	*IGBP1, VEGFA, SNCA*
Negative Regulation Of Cysteine-Type Endopeptidase Activity	3/53	0,000447	956	*IGBP1, VEGFA, SNCA*
Positive Regulation Of Type II Interferon Production	3/58	0,000504	1500	*IL23R, HLA-A, LGALS9*
Response To Molecule Of Bacterial Origin	3/69	0,000746	5158	*IL23R, LGALS9, SNCA*
Regulation Of Cysteine-Type Endopeptidase Activity Involved In Apoptotic Process	3/84	0,001069	5158	*IGBP1, VEGFA, SNCA*
Response To Interleukin-1	3/85	0,001069	429	*IGBP1, LGALS9, SNCA*
Regulation Of Type II Interferon Production	3/87	0,001069	1287	*IL23R, HLA-A, LGALS9*
Positive Regulation Of Peptidyl-Serine Phosphorylation	3/89	0,001069	3581	*TFRC, VEGFA, SNCA*

The late Erythroid cell gene cluster spanned Poly Eb, Ortho Eb, and ARG1+ Ortho Eb, and included *LGALS3, LGALS1, S100A9, FCN1, CXCR4*, and *CXCL8* genes that were overrepresented in the “Antimicrobial Humoral Response”, “Response To Lipopolysaccharide”, and “Response To Molecule Of Bacterial Origin” GO BP terms (**Figure 4E**, **Table 4**).

**Table 4:** Gene Ontology Biological Process overrepresentation analysis of the immunity-related genes with the detected expression in human adult bone marrow late Erythroid cells.

Gene Ontology Biological Process Term	Overlap	*Q*-value	Score	Genes
Response To Molecule Of Bacterial Origin	4/69	0,000043	2209	*CXCL8, LGALS9, S100A9, SNCA*
Antimicrobial Humoral Response	4/100	0,000096	1355	*CXCL8, HLA-A, GAPDH, S100A9*
Response To Lipopolysaccharide	4/159	0,000408	731	*CXCL8, LGALS9, S100A9, SNCA*
Receptor Internalization	3/51	0,000413	1537	*CXCL8, TFRC, SNCA*
Antimicrobial Humoral Immune Response Mediated By Antimicrobial Peptide	3/65	0,000612	1118	*CXCL8, GAPDH, S100A9*
Neutrophil Chemotaxis	3/70	0,000612	1015	*LGALS3, CXCL8, S100A9*
Inflammatory Response	4/236	0,000612	427	*CXCL8, CXCR4, LGALS9, S100A9*
Granulocyte Chemotaxis	3/73	0,000612	960	*LGALS3, CXCL8, S100A9*
Neutrophil Migration	3/77	0,000634	895	*LGALS3, CXCL8, S100A9*
Response To Type II Interferon	3/80	0,000634	851	*LGALS9, GAPDH, SNCA*
Regulation Of Dendritic Cell Differentiation	2/10	0,000634	4974	*LGALS3, LGALS9*
Response To Interleukin-1	3/85	0,000691	786	*CXCL8, LGALS9, SNCA*

We also observed gene expression of other important immunosuppressive gene, that was only expressed at a single stage of differentiation - *C10orf54* gene [encodes for the VISTA protein - an inhibitory immune checkpoint molecule for human T-cells (40)] was expressed at a high level in Baso Eb (**Figure 4A**).

**Figure 5:**
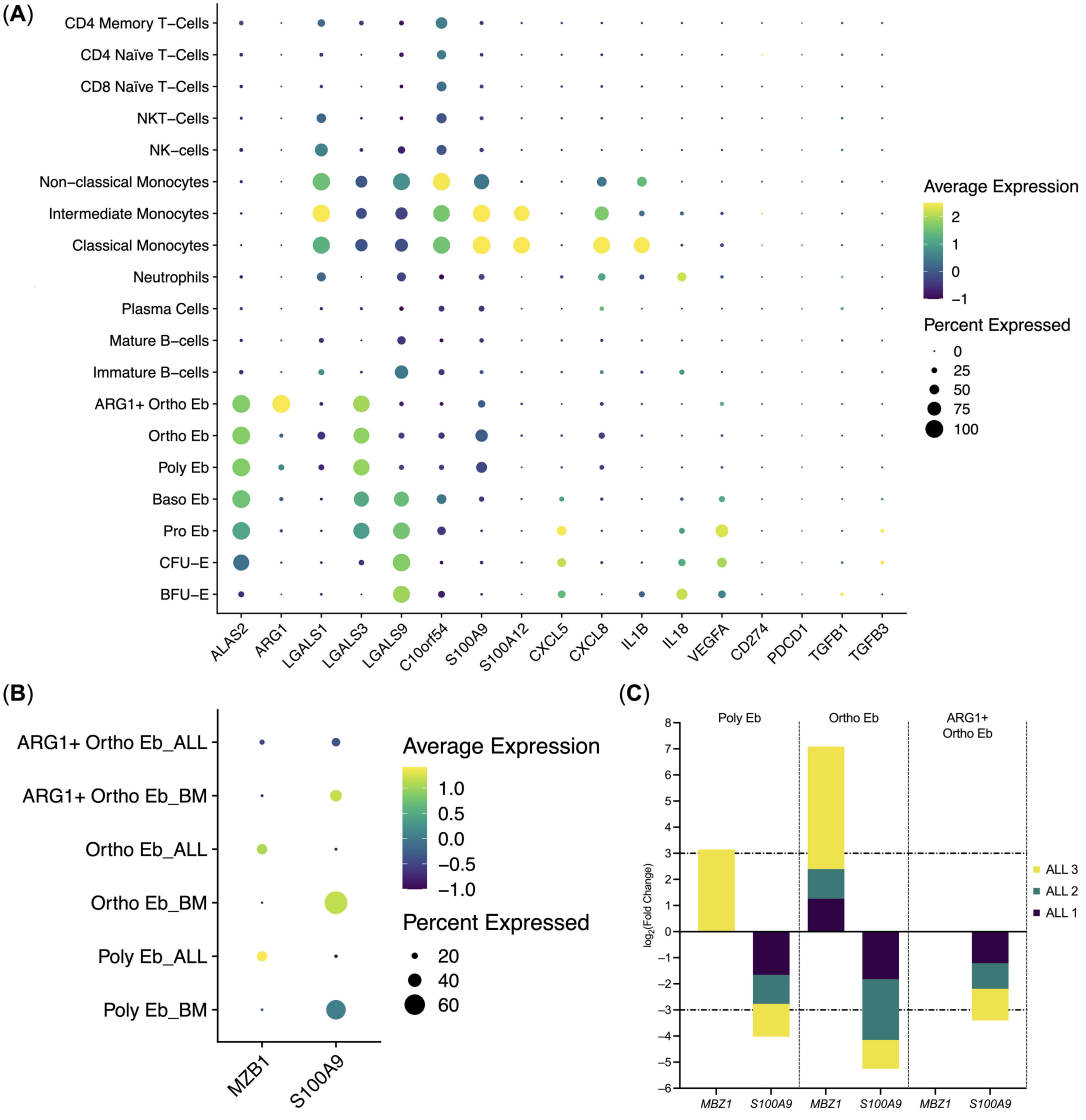
Erythroid cell gene expression in the context of the whole bone marrow and differential gene expression between the ALL Erythroid cells (*n* = 3) and normal Erythroid cells (*n* = 4). **(A)** Dot plot of the Erythroid cell immunity-related genes in the context of the bone marrow mononuclear cells, mean marker expression values were Z-score transformed, the deep purple color represents the lowest marker expression whereas the yellow color represents the maximum marker expression, dot size represents the percentage of Erythroid cells positive for the marker; **(B)** Dot plot of the differentially expressed genes in ALL Erythroid cells, mean marker expression values were Z-score transformed, the deep purple color represents the lowest marker expression whereas the yellow color represents the maximum marker expression, dot size represents the percentage of Erythroid cells positive for the marker; **(C)** Stacked bar plots of the log2(Fold Change) values per differentially expressed gene per cluster, horizontal lines represent log2(Fold Change) thresholds of significance.

We then decided to compare the immunity-related gene expression in normal human bone marrow Erythroid cells with that of the other normal human bone marrow mononuclear cells: *ARG1* gene expression was unique to the ARG1+ Ortho Eb and *CXCL5* gene expression was unique to the early Erythroid cells (BFU-E, CFU-E, Pro Eb, Baso Eb) among the normal human bone marrow mononuclear cells. *IL18* gene expression in BFU-E was not statistically different from Neutrophils (**Figure 5A**).

We also observed that Erythroid cells bear some resemblance with Classical Monocytes in the composition of the expressed immunity-related genes, albeit at different levels: *C10orf54*, *LGALS1*, *S100A9*, *CXCL8*, *IL1B* gene expression was significantly higher in Classical Monocytes, whereas *LGALS3*, *LGALS9*, and *VEGFA* gene expression was significantly higher in Erythroid cells. *C10orf54* gene expression in Erythroid cells was also significantly lower than in T-cells (See **Supplementary Data Sheet 2** for the whole set of differentially-expressed genes).

The expression of the *CD274* (PD-L1), *IL10*, and *PDCD1* (PD-1) genes was not detected in the human adult bone marrow Erythroid cells. Only a minuscule *TGFB1* gene expression was detected in BFU-E and CFU-E, but not in the proper Erythroid cells, i.e., Pro Eb, Baso Eb, Poly Eb, Ortho Eb, ARG1+ Ortho Eb (**Figure 5A**). The *ARG2* gene, another popular target in erythroid cell studies, was not included in the “Immune Response” panel.

We also performed differential gene expression analysis between the adult human ALL Erythroid cells and the adult normal Bone Marrow Erythroid cells and observed the significant down-regulation of the *S100A9* antibacterial immunity gene expression in the late Erythroid cells (Poly Eb, Ortho Eb, ARG1+ Ortho Eb); and the significant up-regulation of the MZB1 gene expression in the Ortho Eb (**Figures 5B, C**). We performed differential gene expression analysis between other normal and ALL bone marrow cell populations but found no ALL-common differentially expressed genes (See **Supplementary Data Sheet 2**).

## 4 Discussion

In this study, we performed a multi-omic analysis of adult human bone marrow Erythroid cells in normal conditions and during acute lymphoblastic leukemia. We have observed the presence of a plethora of immunoregulatory genes in human Erythroid cells, that were overrepresented in MHC Class II antigen presentation and antimicrobial immunity biological process gene signatures, as well as genes that were shown to drive immunosuppression, such as *C10orf54* (VISTA) and *ARG1* (Arginase 1).

We only found MHC Class II antigen presentation genes in early erythroid progenitors, such as BFU-E and CFU-E, but we did not detect any valid MHC Class II alpha and beta chains at these stages of differentiation, thus rendering the exogenous antigen presentation pathway defective in adult human bone marrow Erythroid cells, thus no exogenous antigen presentation is expected by Erythroid cells. The observed antimicrobial gene signature spanned both early Erythroid cells (BFU-E, CFU-E, Pro Eb, Baso Eb) and late Erythroid cells (Poly Eb, Ortho Eb, ARG1+ Ortho Eb) and formed a bipartite complex, thus predicting a possible role for Erythroid cells in innate antimicrobial immunity.

We were also able to narrow down the spectrum of cytokines, expressed by the human bone marrow Erythroid cells: *CXCL5*, *CXCL8*, *IL1B*, *IL18*, and *VEGFA* - two chemokines, two proinflammatory, and a single angiogenic cytokine. We have also previously found *MIF* chemokine gene expression using bulk RNA profiling (41). As *IL1B*, *IL18*, and *VEGFA* were mainly expressed in BFU-E and CFU-E, which comprised 3.5-12.7% of the studied Erythroid cells, their absolute protein expression is expected to be low and chemokines might be the main cytokine product of human bone marrow Erythroid cells as they are expressed at every stage of differentiation. Hypothetically, Erythroid cells could restrict granulocytes to the bone marrow via secretion of the CXCL5 (42) and CXCL8 (IL-8) (43) chemokines.

We can also predict that late Erythroid cells can potentially combat pathogens via Calprotectin. We have previously observed Calprotectin (*S100A8* and *S100A9*) gene expression in Erythroid cells via the bulk RNA profiling of both human (41) and murine erythroid cells (44), and the data in this manuscript allows us to map the Calprotectin gene to the late Erythroid cells, as only they have *S100A9* gene expression among all Erythroid cells.

We also observed that *S100A9* gene expression was down-regulated in the ALL-bone marrow Erythroid cells compared with normal bone marrow Erythroid cells, which could indicate a potential loss of Calprotectin production by bone marrow Erythroid cells at the state of ALL by Erythroid cells.

Another gene that was differentially expressed in the Acute Lymphoblastic Leukemia late Erythroid cells was *MZB1*. As no expression of this gene was detected in normal BM Erythroid cells, detected *MZB1* in late (CD36-negative, CD235-positive) bone marrow Erythroid cells could be potentially indicative of an ongoing ALL.

In the context of all bone marrow mononuclear cells, erythroid cells had the most resemblance in their immune transcriptome with the classical monocytes due to the expression of the antimicrobial and immunoregulatory genes, which means that Erythroid cells express myeloid genes even in the case of the steady-state erythropoiesis.

We detected gene expression of immunosuppressive genes *ARG1*, *LGALS1* (45), *LGALS3* (46), *LGALS9* (47), and *C10orf54* (VISTA) in Erythroid cells. Arginase 1 was the main point of interest in this study, as it formed its own stage of Erythroid cell differentiation - ARG1+ Ortho Eb, which was separate from the *ARG1*-negative classic Ortho Eb. ARG1+ Ortho Eb were also separate from the main branch of erythroid cell differentiation. Expression of the *ARG1* gene was also the main branching driver that split Poly Eb into *ARG1*-negative Ortho Eb and *ARG1*-positive ARG1+ Ortho Eb. The molecular basis of this split of Poly Eb in either ARG1-positive or ARG1-negative Ortho Eb still requires further investigation. Moreover, ARG1+ Ortho Eb was the only cell population among the normal bone marrow mononuclear cells to express the *ARG1* gene. Other important immunoregulatory genes *LGALS1*, *LGALS3*, *LGALS9*, and *C10orf54* (VISTA) all split among different stages of Erythroid cell differentiation, which means that Erythroid cells pose immunosuppressive potential at every stage of their differentiation.

Unlike the solid tumor-induced Erythroid cells (48, 49), both normal and ALL bone marrow Erythroid cells did not express either *PDCD1* (PD-1) or *CD274* (PD-L1) genes, which indicates a difference in the gene expression profile of immunoregulatory molecules between normal bone marrow Erythroid cells and their solid tumor-induced counterparts. We also did not find any *IL10* gene expression in human bone marrow Erythroid cells that was previously described for human fetal liver Erythroid cells (50), which can indicate the presence of tissue-dependent cytokine gene expression profiles for erythroid cells.

As for the *TGFB1* gene, its expression was almost undetectable in the human bone marrow Erythroid cells, which is unlike murine bone marrow Erythroid cells, where *TGFB1* was one the most expressed genes overall in a similar immune response gene panel (44).

In conclusion, we performed a thorough investigation of steady-state erythropoiesis/normal adult human bone marrow Erythroid cells using targeted proteomics and transcriptomics via the CITE-seq protocol and found that such Erythroid cells express *ARG1*, *LGALS1*, *LGALS3*, *LGALS9* and *VISTA* immunosuppressive genes, *CXCL5* and *CXCL8* chemokines and that such Erythroid cells express antimicrobial immunity and MHC Class II antigen presentation genes without any myeloid-transdifferentiating stimulus.

## Data Availability

The datasets presented in this study can be found in online repositories. The names of the repository/repositories and accession number(s) can be found below: GSE261733 (GEO). The original codes presented in the study are publicly available. This data can be found here: https://github.com/Perik-Zavodskii/ALL-BM-scMultiomics-BD-Rhapsody.
