# Supplementary material for: Single-cell multi-omics reveal stage of differentiation and trajectory-dependent immunity-related gene expression patterns in human erythroid cells
Source: Front Immunol. 2024 Aug 29;15:1431303. doi: 10.3389/fimmu.2024.1431303 (PMC11390661; doi:10.3389/fimmu.2024.1431303)
Supplement: Supplementary file 1 [file DataSheet1.docx]

Supplementary Material


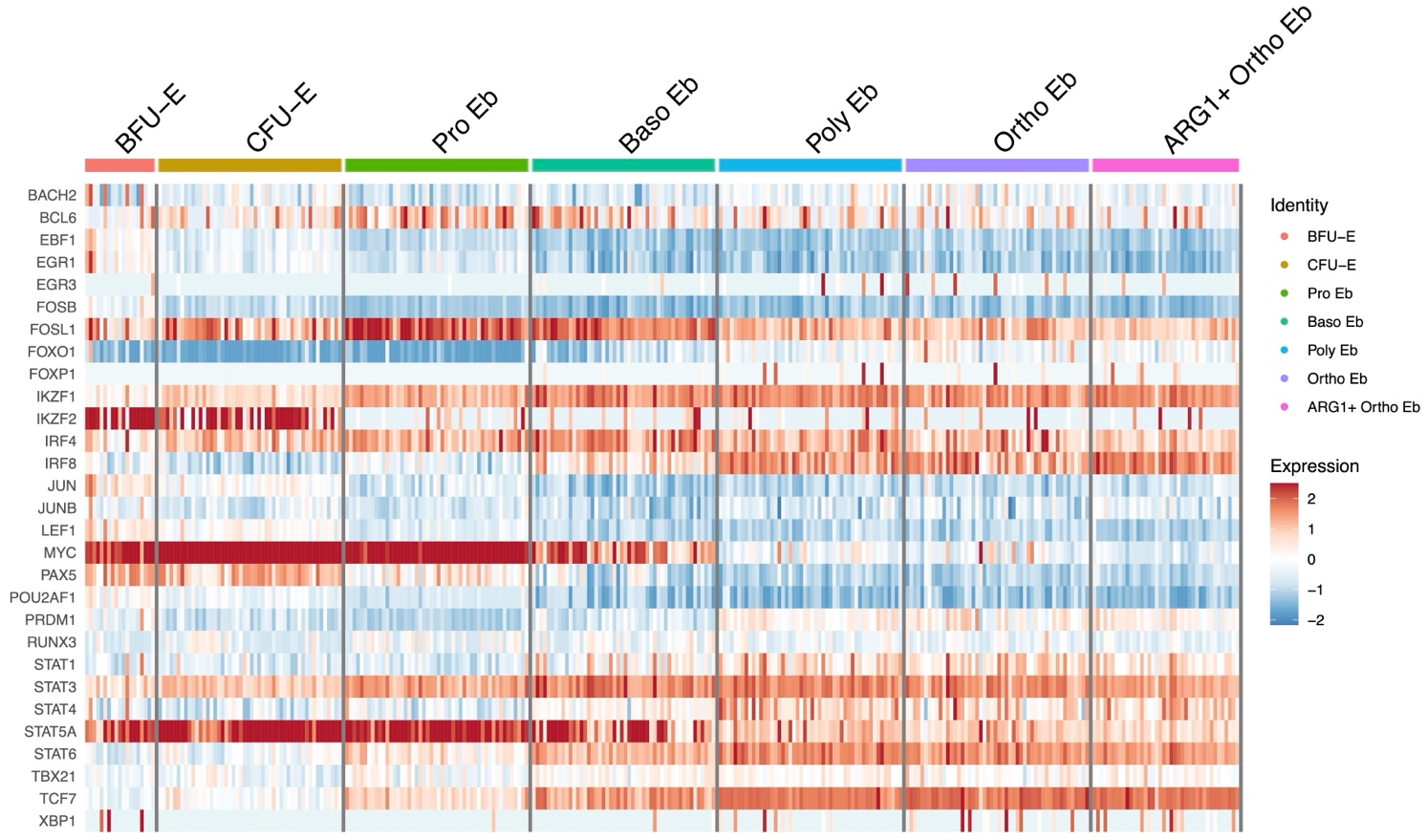


Supplementary Figure 1. Heat map of the Z-score-standardized AUC scores of transcription factor activity per normal bone marrow Erythroid cell cluster obtained by pySCENIC, each line represents a cell.
